# Rosemary Essential Oil as a Natural Additive in Food Industry: Recent Developments in Encapsulation Techniques

**DOI:** 10.3390/foods15050893

**Published:** 2026-03-05

**Authors:** Pavle Simić, Nataša Poklar Ulrih

**Affiliations:** Biotechnical Faculty, University of Ljubljana, Jamnikarjeva ulica 101, 1000 Ljubljana, Slovenia

**Keywords:** essential oil, encapsulation, antioxidant, antimicrobial, 1,8-cineole

## Abstract

Rosemary essential oil (REO) is a complex mixture of volatile organic compounds (VOCs), predominantly oxygenated monoterpenes such as 1,8-cineole, camphor, and borneol, which together exhibit antioxidant and antimicrobial activities. These properties make REO a promising natural alternative to synthetic food additives; however, its high volatility, low water solubility, chemical instability, and intense aroma significantly limit its direct application in food systems. Encapsulation has therefore emerged as a key strategy to enhance REO stability, preserve bioactivity, and enable controlled release while reducing sensory impact. This review critically examines conventional and advanced techniques for REO encapsulation. These techniques are comparatively evaluated by addressing their advantages and limitations, with particular emphasis on wall material selection and its role in controlling release behaviour and functional performance in real food matrices. In addition to summarising current applications in food preservation, functional ingredients, and active packaging, this review highlights a key research gap: the limited post-encapsulation characterisation of REO chemical composition, especially minor VOCs responsible for synergistic biological effects. Addressing this gap is essential for the design of encapsulation systems that effectively integrate aroma, preservation, and functionality in clean-label food products.

## 1. Introduction

Rosemary (*Salvia rosmarinus* Spenn formerly known as *Rosmarinus officinalis* L.), a perennial aromatic shrub native to the Mediterranean region, is widely cultivated worldwide due to its adaptability to drought, poor soils, and warm climates [1]. Traditionally valued as a culinary herb, rosemary has also played a prominent role in folk medicine for its digestive, antimicrobial, and preservative properties [2]. These effects are largely attributed to its diverse secondary metabolites, including phenolic diterpenes, phenolic acids, and volatile terpenoids, which contribute to both plant defence and biological activity [3,4]. Beyond its culinary uses, rosemary has attracted significant scientific and industrial interest as one of the richest natural sources of antioxidant compounds among aromatic plants. While non-volatile phenolics such as carnosic acid and rosmarinic acid dominate rosemary extracts, the volatile fraction—rosemary essential oil (REO)—also exhibits notable antioxidant, antimicrobial, and sensory properties [4]. The composition of REO is highly variable, influenced by genetic factors, environmental conditions, and post-harvest processing [5,6].

REO is primarily obtained by steam distillation of aerial plant parts and consists of a complex mixture of monoterpenes and oxygenated monoterpenes, including 1,8-cineole, camphor, α-pinene, and borneol [7]. This chemical diversity underpins its multifunctionality, enabling applications as a flavouring agent, natural preservative, and functional ingredient in food, pharmaceutical, and cosmetic products. However, its high volatility and susceptibility to degradation by light, heat, and oxygen limit direct use, prompting research into stabilisation and encapsulation strategies. The growing demand for clean-label, natural products has intensified interest in essential oils (EOs) as alternatives to synthetic additives. REO, in particular, stands out for its potent antioxidant, antimicrobial, and anti-inflammatory activities, making it a promising candidate for health-oriented food formulations [8]. Nevertheless, its strong aroma and physicochemical instability pose challenges for incorporation into food systems. Encapsulation has emerged as an effective approach to enhance REO stability, control release, and preserve bioactivity during processing and storage [9,10,11]. Techniques range from conventional methods such as spray drying to advanced systems including nanoemulsions, liposomes, and solid lipid nanoparticles, with wall materials and process parameters critically influencing performance.

This review provides a comprehensive overview of the chemical composition, bioactive properties, and encapsulation strategies of REO. To the best of our knowledge, no previous review has systematically and in detail compared different encapsulation techniques while critically discussing their advantages, limitations, and potential applications for encapsulated REO. Furthermore, this review highlights the benefits of encapsulation and examines current and emerging applications of encapsulated REO in the food industry, aiming to guide future research and support the industrial adoption of EOs as natural additives.

## 2. Methodology of the Review

In order to obtain a comprehensive and up-to-date overview, a systematic approach was adopted in collecting and analysing the literature. This section describes the methods used to identify, select and synthesise relevant scientific information on encapsulation techniques, chemical composition and applications of REO in the food industry.

### 2.1. Search Strategy

The literature search was conducted using major scientific database Web of Science and Scopus, between September and December 2025. The following keywords and Boolean operators were applied in various combinations for title, abstract and keywords:

- (“rosemary essential oil” OR “*Rosmarinus officinalis* essential oil” OR “*Salvia rosmarinus Spenn* essential oil”) AND (encapsulat* OR microencapsulat* OR nanoencapsulat*)

Additionally, backward citation tracking was performed to identify relevant articles cited in high-impact reviews and research papers.

### 2.2. Inclusion, Exclusion Criteria, Data Extraction and Organisation

**Figure 1:**
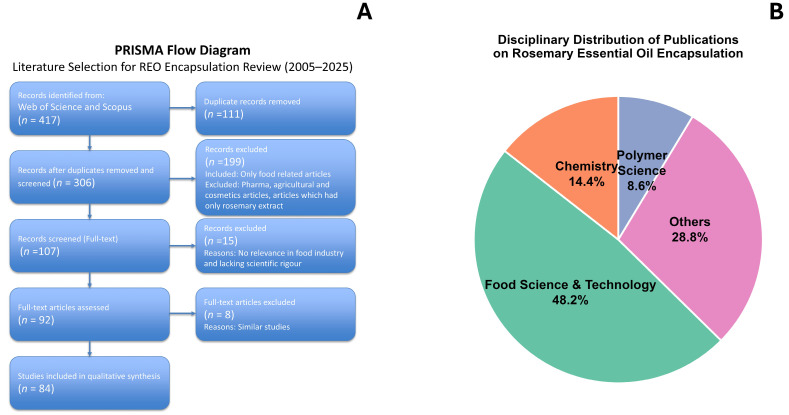
(**A**) Prisma flow diagram—selection process for review; (**B**) disciplinary distribution of publications on REO encapsulation.

The articles were included in this review based on specific inclusion criteria to ensure relevance and scientific quality. The review framework was developed in accordance with the Preferred Reporting Items for Systematic Reviews and Meta-Analyses (PRISMA) guidelines [12]. Figure 1A presents the PRISMA flow diagram illustrating the study selection process, including identification, screening, exclusion, and final inclusion of studies for analysis. Eligible studies were published in peer-reviewed journals between 2005 and 2025 and focused explicitly on techniques used for encapsulation of REO and its application in the food industry. To ensure systematic, duplicate-free inclusion, articles meeting these requirements were exported and managed with Zotero (7.0.32). After removing duplicates, both original research articles and reviews were considered. A high number (*n* = 306) of studies were found to be related to REO and encapsulation techniques. It was noted that many articles included the chemical composition and biological activities of REO before encapsulation. In the second step, after checking titles, abstracts, and keywords, studies focusing on EOs other than REO and those limited to pharmacological or cosmetic applications with no food-related relevance were excluded based on the exclusion criteria. Studies lacking scientific rigour were also excluded, reducing the number of articles to *n* = 107. In the final step, the full texts were analysed to verify eligibility and relevance to the scope of this review. The final number of studies included was 84. This selection strategy ensured a targeted and reliable data set for comprehensive analysis. Additional references cited in this review include biosynthesis of VOCs in REO and regulatory documents that were not part of the systematic screening process.

Most of the research is conducted in the fields of food science and technology, applied chemistry and polymer science (Figure 1B). Other contributions come from the disciplines of chemical engineering, agronomy and physical chemistry. This field of study is interdisciplinary, as the distribution across the various disciplines shows. These results indicate that the food, chemical and pharmaceutical sectors are beginning to recognise the growing importance of REO encapsulation.

## 3. Chemical Composition of REO

**Figure 2:**
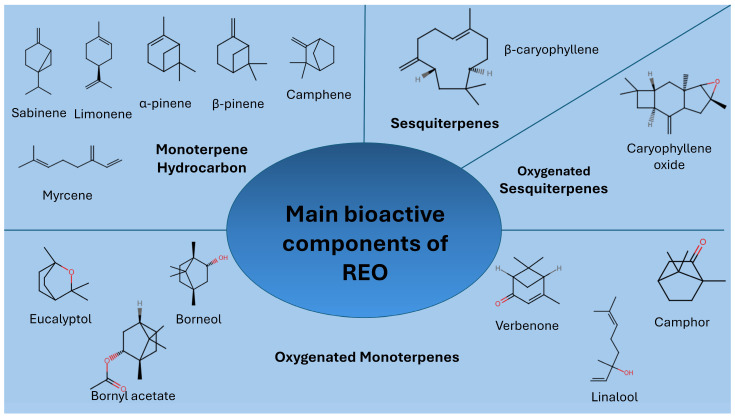
Main bioactive components of REO.

REO belongs to the family of *Lamiaceae* EOs and is rich in volatile organic compounds (VOCs) [13]. The main groups of compounds which are present are monoterpene hydrocarbons, oxygenated monoterpenes and sesquiterpenes in small amounts (Figure 2). These constituents can range significantly in concentration, depending on factors such as plant genotype or chemotype [14], geographical origin and climate [6] or extraction technique and parameters [15]. From Table 1, it can be observed that the chemical composition of REO is influenced by both the plant part used and the type of distillation method applied. Additionally, variations associated with the country of origin are evident.

Based on the most abundant compound in REO there are several different chemotypes. Ben Abada et al. (2019) [25] investigated Tunisian REOs, which showed three different chemotypes: (1) 1,8-cineole/camphor/a-pinene, (2) 1,8-cineole/camphor and (3) camphor/1,8-cineole. Similar results were reported by [16] and Ben Jemia et al. (2015) [26], reporting the occurrence of three chemotypes. On the other hand, Napoli et al. (2010) [27] classified REOs into three chemotypes: (1) cineoliferum (high in 1.8-cineol), (2) camphoriferum (camphor >20%) and (3) verbenoniferum (verbenone >15%). Nevertheless, Satyal et al. (2017) [14] reported the presence of five chemotypes: (1) a-pinene/1,8 cineol, (2) verbenone/a-pinene/camphor/1,8 cineol, (3) myrcene/1,8 cineol/camphor, (4) 1,8 cineol/camphor/a-pinene, and (5) a-pinene/b-pinene/camphene.

In the literature, the most frequently occurring chemotypes can be found where 1,8-cineole is most abundant. 1,8-Cineole is a monoterpene oxide known for its anti-inflammatory, antioxidant, antimicrobial and anticancer activity [28]. 1,8-Cineole is formed by the cyclisation of GPP via the enzyme 1,8-cineole synthase, also called cineole cyclase [29]. Camphor is a ketone with notable antibacterial, antifungal, antioxidant, anticancer, analgesic, and anti-inflammatory properties [30]. It is also like 1,8-cineole biosynthesised via the MEP pathway in the chloroplasts. In nature, α-pinene is biosynthesised by α-pinene synthase, which cyclises GPP into the pinane skeleton [31,32]. β-Pinene is the isomer of α-pinene and is typically derived from the same pinene synthase activity that yields a mixture of α- and β-pinene. Limonene is a monocyclic monoterpene produced by limonene synthase from GPP—this enzyme forms a cyclohexenyl cation and deprotonates to limonene. While limonene is a minor fraction in REO, it is an important intermediate in citrus EOs [33]. Interestingly, limonene synthase genes have also been identified in rosemary which are often co-expressed with cineole synthase due to the common precursor [34]. Verbenone, borneol, and linalool are oxygenated terpenes contributing to the aroma of REO with biological activity [35].

## 4. Biological Activities of REO

The biological activities of REO result from its complex and chemically diverse profile of VOCs, whose functionality depends not only on their presence but also on their relative proportions, chemical structures, and interactions. Unlike single-molecule additives, REO functions as a multicomponent bioactive system, in which both major and minor constituents contribute to overall activity through complementary and synergistic mechanisms. REO has been extensively reported to exhibit antioxidant, antimicrobial, and anti-inflammatory activities, making it a promising natural preservative and functional ingredient for food applications. However, these biological effects cannot be attributed to individual compounds alone. Instead, they reflect the combined action of oxygenated monoterpenes, monoterpene hydrocarbons, and minor VOCs, which influence redox behaviour, microbial membrane integrity, and oxidative stability in complex food matrices.

### 4.1. Antioxidant Activity

The antioxidant behaviour of REO results from multiple, compound-specific reaction mechanisms rather than a single dominant pathway [36]. In EOs, antioxidant activity is typically manifested through radical scavenging, chain termination, co-oxidation modulation, and metal interaction, depending on the chemical structure of the volatile organic compounds involved [37]. Oxygenated monoterpenes containing hydroxyl groups—such as linalool, α-terpineol, and borneol—can act as weak hydrogen atom donors. These compounds can transfer a hydrogen atom to peroxyl or alkoxyl radicals, thereby terminating radical chain reactions in lipid oxidation systems. Although their hydrogen-donating capacity is lower than that of phenolic antioxidants, their relatively high volatility and mobility enable effective interaction with radical species, particularly in the early stages of oxidation. At low concentrations, this mechanism contributes positively to antioxidant activity by slowing radical propagation and stabilising reactive intermediates [38,39].

Some monoterpenes do not act as classical radical scavengers but instead modify oxidation kinetics through co-oxidation mechanisms. In these cases, the compound reacts preferentially with radicals, forming relatively unreactive products that reduce the overall rate of lipid peroxidation [40]. This termination-enhancing behaviour has been proposed for compounds such as linalool and α-terpineol, particularly when present at low concentrations where reaction products do not initiate further radical formation. At higher concentrations, the same compounds may exhibit pro-oxidant behaviour. Excess linalool or other oxygenated monoterpenes can undergo oxidation to form reactive intermediates, such as hydroperoxides or aldehydes, which may re-initiate radical chains. In addition, high concentrations can increase oxygen solubility or alter microenvironmental polarity in lipid systems, facilitating oxidative reactions rather than suppressing them. Major REO constituents such as 1,8-cineole, camphor, α-pinene, and β-pinene generally lack functional groups capable of direct radical scavenging [40,41]. Their antioxidant contribution is therefore indirect. These compounds may influence antioxidant performance by altering the diffusion of reactive species, modifying lipid phase behaviour, or stabilising reactive intermediates through physical interactions. Importantly, such compounds can participate in synergistic networks, where their presence enhances the effectiveness of minor oxygenated monoterpenes. This synergy explains why whole REO often exhibits stronger antioxidant activity than mixtures of isolated compounds, even when major constituents show little activity individually.

### 4.2. Antimicrobial Activity

Alongside the antioxidant activity of EOs, antimicrobial activity is one of the most studied properties, which is important both for the preservation of food and for combating diseases of microbial origin in humans and animals. The antimicrobial activity of REO is mainly determined by the chemical structure, lipophilicity, volatility, and membrane affinity of its VOCs. In contrast to antioxidant activity, which is strongly concentration-dependent and may show pro-oxidant effects at high concentrations, antimicrobial efficacy generally increases with concentration but remains highly dependent on synergistic interactions among multiple constituents. The primary mechanism of antimicrobial action of REO is the physical disruption of microbial cell membranes. Lipophilic monoterpenes, such as α-pinene, β-pinene, myrcene, 1,8-cineole, camphor, and borneol, readily partition into the lipid bilayer of bacterial and fungal membranes [3,5,42]. Components of EOs disrupt microbial cell membranes, inhibiting the growth of common foodborne pathogens such as *E. coli*, *Listeria monocytogenes*, and *Staphylococcus aureus* [43]. This interaction increases membrane fluidity and permeability, resulting in leakage of intracellular components, loss of proton motive force, and impairment of essential metabolic functions. Oxygenated monoterpenes generally have stronger membrane-disruptive effects than hydrocarbon terpenes because they can interact with both hydrophobic lipid chains and polar head groups. In the study of Okoh et al. [44], it has been demonstrated that the oil extracted via solvent-free microwave extraction exhibited superior antimicrobial activity compared to hydrodistilled oil, likely due to its higher content of oxygenated compounds. Jiang et al. [5] concluded that the antimicrobial activity of REO was stronger than that of its individual components, such as pinene and 1,8-cineole, suggesting that its efficacy may result from a synergistic effect among its constituents. REO exhibited antimicrobial activities against both each of gram-negative (*Escherichia coli*, *S. thyphimurium* and *Klebsiella pneumonia*) and gram-positive (*Staphylococcus aureus* and *S. epidermidis*) bacteria [42]. REO was incorporated into meat, with antibacterial activity reported against *Brochothrix thermosphacta* and *Enterobacteriaceae*, increasing the shelf life of fresh meat [45]. Numerous studies demonstrate that whole REO exhibits stronger antimicrobial activity than its isolated components, highlighting the importance of synergistic interactions [5,38]. Minor VOCs can enhance antimicrobial efficacy by increasing membrane permeability, reducing microbial stress responses, or facilitating the entry of other active compounds [43]. This cooperative behaviour allows effective antimicrobial action at lower individual compound concentrations, reducing sensory impact while maintaining efficacy.

Despite its broad antioxidant and antimicrobial activities, the practical application of REO in food systems remains technologically constrained. Its high volatility leads to rapid evaporation during processing, while exposure to heat, oxygen, and light accelerates oxidative degradation and alters the native VOC profile. Additionally, poor water solubility limits homogeneous dispersion in aqueous matrices, and its intense aroma may exceed sensory acceptance thresholds when applied at biologically effective concentrations. Selective loss or transformation of minor constituents can also disrupt synergistic interactions that underpin REO’s functional efficacy. These physicochemical and sensory limitations highlight the need for stabilisation strategies capable of preserving chemical integrity, modulating release behaviour, and reducing sensory impact, thereby motivating the development of targeted encapsulation approaches.

## 5. Encapsulation Techniques

**Figure 3:**
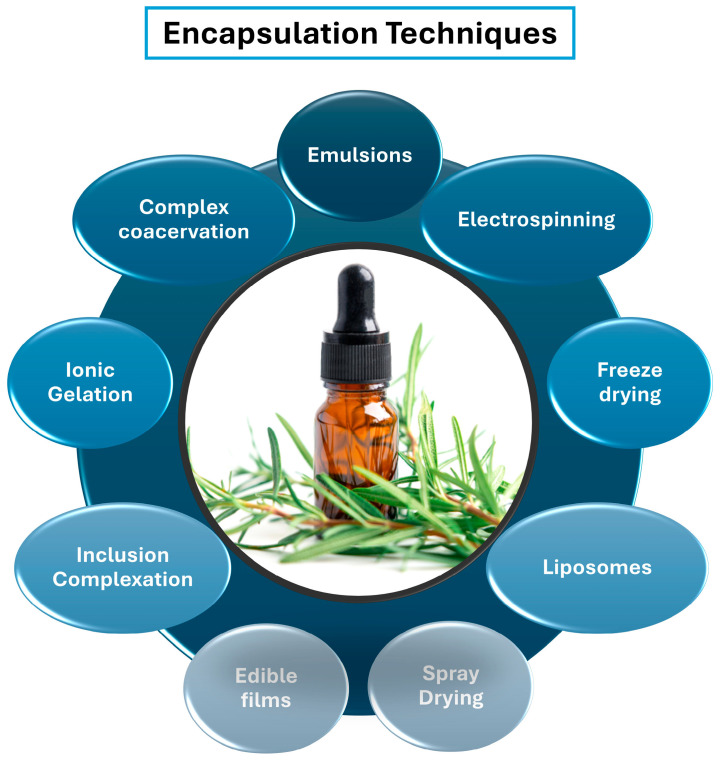
Techniques used for encapsulation of REO.

REO is characterised by high volatility, susceptibility to oxidation, limited water solubility, and a strong sensory profile, all of which restrict its direct application in food systems [46]. Encapsulation serves as a functional preservation approach, designed to stabilise the native VOC composition of REO, limit selective loss of minor compounds, and modulate release behaviour within food matrices. Inappropriate processing conditions can disrupt this balance through volatilisation, oxidation, or thermal degradation, leading to reduced biological efficacy even when total oil retention appears high. A wide range of encapsulation techniques—from conventional microencapsulation methods to advanced nano- and micro-based systems—have been developed to address these challenges (Figure 3). These techniques differ substantially in their operating conditions, encapsulation efficiency, ability to retain volatile compounds, release kinetics, scalability, and compatibility with food applications. Consequently, the choice of encapsulation strategy must consider not only process efficiency and cost, but also preservation of VOC composition and functional performance in the target food system.

### 5.1. Spray Drying

Spray drying is a widely used technique for microencapsulating EOs to increase its stability, protect it from environmental influences and facilitate its incorporation into various products. In this process, the REO is dispersed in a solution of wall materials and then atomised and rapidly dried to produce stable microcapsules. Commonly used wall materials for spray drying encapsulation include maltodextrin, gum arabic, whey protein isolate and inulin, either individually or in combination, due to their excellent film-forming and emulsifying properties [11,47]. The selection of a suitable wall material for microencapsulation by spray drying plays a crucial role in achieving high encapsulation efficiency and ensuring the stability of the resulting microcapsules. The selection process usually depends on various physicochemical properties, including solubility, molecular weight, glass transition or melting point, degree of crystallinity, diffusivity and the ability of the material to form films and stabilise emulsions [48]. In the study by Fernandes et al. [49], REO was encapsulated by spray drying using different combinations of gum arabic, maltodextrin, modified starch, and inulin. Higher concentrations of 1,8-cineole were retained after spray drying in these wall materials, which may explain the observed antimicrobial activity against mesophilic bacteria in cheese. In addition, encapsulation using a mixture of modified starch and maltodextrin resulted in high encapsulation efficiency and effective retention of volatile compounds, whereas the incorporation of inulin increased the moisture content of the resulting powders. The advantages of spray drying for the encapsulation of REO include improved oxidative stability and the potential for controlled release of active ingredients. However, the technique also has some limitations, such as the possibility of thermal degradation of heat-sensitive components due to the high inlet temperatures and the need for careful optimisation of process parameters to achieve high encapsulation efficiency and product quality [50,51].

### 5.2. Freeze Drying

Freeze-drying, also known as lyophilisation, is a gentle dehydration technique often used for the encapsulation of sensitive bioactive compounds such as EOs [52]. This method involves the removal of water by sublimation at low temperature and under vacuum, which preserves the structural and chemical integrity of the volatile components. As no heat is generated during freeze-drying, the risk of thermal degradation and oxidation is significantly reduced, making it particularly suitable for EOs with sensitive flavour profiles. Typically, wall materials such as proteins, polysaccharides or gums are used to form stable matrices that trap the oils prior to drying. Although it is a time- and energy-consuming process, freeze drying remains an extremely effective method for producing stable, high quality encapsulated EOs, especially for applications that require maximum preservation of functionality. In a recent study, REO was successfully encapsulated in zein nanoparticles using the freeze-drying technique [53]. The resulting nanoparticles exhibited sizes ranging from 70 to 200 nm, as determined by scanning electron microscopy, and showed an encapsulation efficiency of about 71%, indicating the effectiveness of this method in stabilising REO for potential applications in the food industry. Turasan et al. [54] successfully encapsulated REO using freeze-drying, with whey protein concentrate and maltodextrin serving as wall materials. The optimal formulation, featuring a whey protein-to-maltodextrin ratio of 3:1 and a core-to-coating ratio of 1:20, achieved high encapsulation efficiency and enhanced the stability of 1,8-cineole, a key component of REO. Also, REO microcapsules obtained by freeze drying were then incorporated paper coatings that exhibited antimicrobial activity against *E. coli*, indicating their potential application in active food packaging [55]. In a comparative study of different encapsulation techniques, it was found that although freeze drying achieves the highest encapsulation yield, it has the lowest encapsulation efficiency and produces particles with high porosity, which may be advantageous for applications requiring rapid release [56].

### 5.3. Emulsions

Emulsification is a widely used technique for encapsulating EOs, providing protection from environmental influences such as oxidation, light and heat while increasing their stability and bioavailability. In this method, EOs are dispersed in a continuous aqueous phase to form oil-in-water emulsions that are stabilised by emulsifiers or surfactants and can be further processed into micro- or nano-sized droplets. Nanoemulsions are emulsions in the submicron range that offer improved stability, bioavailability and controlled release of encapsulated substances. REO is known for its antioxidant and antimicrobial properties and has been effectively encapsulated in nanoemulsion systems to improve its application in various industries. Addition of REO nanoemulsions to dairy products such as pasteurised cream and Karish cheese has shown significant antimicrobial activity against pathogens such as *Listeria monocytogenes* and *Aspergillus flavus*, while improving oxidative stability and sensory properties [57]. In animal nutrition, dietary supplementation with nano-encapsulated REO has shown promising results. A study with broiler chickens showed that the addition of 200 mg/kg nano-encapsulated REO to the feed improved body weight gain, feed efficiency, nutrient digestibility and meat quality [58].

### 5.4. Inclusion Complexation

Inclusion complexation with cyclodextrins (CDs) has emerged as a promising strategy for the encapsulation of EOs, especially REO, to improve their stability, solubility and controlled release properties [59]. CDs are cyclic oligosaccharides characterised by a hydrophobic central cavity and a hydrophilic outer surface, which allows them to form host–guest complexes with hydrophobic molecules such as the constituents of REO. Among the different types of CDs, *β*-cyclodextrin (*β*-CD) is most commonly used due to its suitable cavity size and cost efficiency [60,61]. In the study by Halahlah et al. [59], methyl-*β*-cyclodextrin exhibited the highest inclusion efficiency and retained the greatest proportion of 1,8-cineole. However, this strong retention was associated with a reduced release rate of REO, which correlated with lower antibacterial activity. In contrast, 2-hydroxypropyl-*β*-cyclodextrin showed a lower affinity for 1,8-cineole but more effectively retained camphor and verbenone. This weaker binding resulted in faster release kinetics and, consequently, stronger antimicrobial activity. The formation of REO-*β*-CD inclusion complexes has been shown to protect volatile components from degradation, improve water solubility and modulate the release profile, thereby improving the bioavailability and efficacy of REO in various applications. The advantages of using cyclodextrin inclusion complexes for the encapsulation of REO include improved water solubility, effective odour masking and protection against oxidative degradation. These benefits contribute to improved stability and bioavailability of REO in various applications. However, challenges associated with this encapsulation method include a relatively lower loading capacity compared to other techniques such as spray drying and the complexity of the preparation steps, which may involve multiple stages and precise control of conditions. Despite these challenges, the use of cyclodextrin inclusion complexes remains a valuable strategy for the stabilisation and delivery of REO.

### 5.5. Liposomes

Liposomes and nanoliposomes are advanced lipid-based delivery systems used to encapsulate REO to improve its stability, bioavailability and controlled release. Liposomes are spherical vesicles consisting of phospholipid bilayers that can encapsulate both hydrophilic and lipophilic compounds [62]. Nanoliposomes, a subclass with nanometric dimensions, offer improved penetration and stability due to their smaller size [63]. Nanoliposomes loaded with REO were incorporated into temperature-sensitive polymer films for the development of active food packaging. These films exhibited a dual release mechanism at certain temperatures, enabling prolonged antioxidant activity and potential applications in food preservation [64]. However, there are limitations to using liposomes for REO encapsulation. They can be unstable in aqueous media, leading to potential leakage or degradation of the encapsulated oil. Moreover, the production of liposomes can be relatively costly, which may hinder large-scale industrial applications. Despite these challenges, ongoing research aims to improve the stability and cost-effectiveness of liposomal encapsulation methods to fully leverage their benefits for EO delivery.

### 5.6. Ionic Gelation

Ionic gelation involves the interaction of charged biopolymers such as alginate, chitosan or gelatine with polyvalent ions to form a gel matrix. In the context of REO encapsulation, calcium ions are commonly used to cross-link negatively charged alginate, resulting in the formation of calcium alginate beads. Berraaouan et al. [65] developed microcapsules of calcium alginate and a calcium alginate/montmorillonite hybrid by ionotropic gelation. These microcapsules exhibited improved encapsulation efficiency (up to 83%) and loading capacity (up to 73%) due to the incorporation of montmorillonite clay, which provides a larger adsorption surface area. In addition, the hybrid microcapsules showed improved thermal stability and a prolonged release profile of REO compared to those without clay, indicating their potential for applications requiring sustained release and increased stability. Similarly, Dolçà et al. [66] encapsulated REO with an alginate and calcium chloride system. Their study showed that the antimicrobial and antifungal properties of REO remained unchanged after encapsulation, emphasising the non-destructive nature of the ionic gelation method. Ionic gelation, particularly using alginate, represents a suitable encapsulation approach for REO in beverage applications. Alginate is already widely used in the drink industry, for example in bubble tea and popping boba, which facilitates its technological compatibility and regulatory acceptance for encapsulating REO.

### 5.7. Complex Coacervation

Complex coacervation is a phase separation process driven by the electrostatic interaction between two or more oppositely charged colloids, usually proteins and polysaccharides. This method facilitates the formation of a coacervate phase that can encapsulate active compounds such as REO. Amani et al. [67] have successfully encapsulated REO in almond gum and gelatine complex coacervates to form colloidal carriers. Their study of gastrointestinal in vitro release and cytotoxicity of the encapsulated REO showed that the encapsulation process preserved the bioactivity of REO while reducing its cytotoxicity, highlighting the suitability of complex coacervation for the development of safe and effective delivery systems. The advantages of complex coacervation include mild processing conditions and excellent preservation of bioactivity, making it suitable for sensitive compounds such as EOs. However, this method also presents challenges, such as batch-to-batch variability and difficulties in controlling particle size, which can affect the consistency and performance of the final product.

### 5.8. Electrospinning

Electrospinning is a technique for producing ultrafine polymer fibres containing bioactive compounds by applying a high-voltage electric field to a polymer solution or melt. Under the applied field, a charged jet is ejected from the needle tip, forming a Taylor cone, and is stretched into continuous fibres with diameters ranging from nanometres to micrometres as the solvent evaporates and the polymer solidifies. The resulting non-woven fibre mats are collected on a grounded surface [68]. Although electrospinning remains primarily at the research stage for food applications, it shows considerable potential for active food packaging, controlled release, and antimicrobial coatings [69]. REO was encapsulated into zein-based electrospun fibres at varying concentrations. The encapsulated fibres demonstrated antimicrobial activity against *S. aureus* and *E. coli*, with release profiles influenced by pH, indicating their potential application in active food packaging systems [10]. In another study, REO was encapsulated into zein-based electrospun fibres to create active edible coatings with antimicrobial properties [70]. These coatings demonstrated enhanced antibacterial effectiveness against *Listeria monocytogenes*, *Staphylococcus aureus*, and mesophilic bacteria on cheese slices, particularly with higher EO concentrations and over extended storage at 4 °C, indicating their potential for improving food safety and shelf life. Amjadi et al. [71] encapsulated REO into electrospun zein nanofibres reinforced with ZnO nanoparticles and κ-carrageenan for antimicrobial food packaging applications. These nanofibres exhibited significant antibacterial activity against *S. aureus* and *E. coli*, strong antioxidant capacity, and no cytotoxicity, highlighting their potential as a biocompatible active packaging material. Electrospinning offers promising prospects for the encapsulation of EOs, especially for the development of multifunctional nanofibre systems with targeted delivery, prolonged release and improved stability. Ongoing advances in the formulation of biopolymers, the scalability of the process and the integration of active ingredients could further expand applications in the areas of food packaging, pharmaceuticals and biomedicine.

### 5.9. Edible Films

The incorporation of EOs into edible films has proven to be an innovative and sustainable strategy to improve food preservation, especially due to their inherent antimicrobial and antioxidant properties. Edible films usually consist of biodegradable biopolymers such as polysaccharides, proteins and lipids. These materials provide a matrix for the controlled release of REO and at the same time act as a physical barrier to oxygen, moisture and microbial contaminants [72,73]. Polysaccharide-based films in particular offer excellent gas barrier properties, while protein-based films have higher mechanical strength. However, both types of films often have poor water barrier properties, which can be improved by adding lipophilic agents such as REO or by combining them with hydrocolloids. The encapsulation of REO in edible films can be achieved by various techniques; one of the best known in the literature is film casting. This method allows uniform dispersion and stabilisation of REO in the film matrix. Yeddes et al. (2020) [74] reported that edible films loaded with REO exhibit increased antimicrobial activity against common foodborne pathogens such as *Bacillus subtilis*, *Staphylococcus aureus*, *Enterococcus aerogenes*, *Enterococcus faecalis* and *Escherichia coli.* REO was encapsulated in a film of nisin and *Lycium barbarum* polysaccharides for active food packaging applications [75]. Film effectively reduced the populations of *S. aureus* and *E. coli* O157:H7 in treated beef without affecting its sensory properties. These functional properties make REO-incorporated films particularly suitable for active packaging systems designed to improve food safety and extend the shelf life of products.

### 5.10. Comparative Assessment of Encapsulation Techniques

Although numerous encapsulation techniques have been successfully applied to REO, no single approach is universally optimal. Benefits, limitations and their industrial scalability of encapsulation techniques used for REO encapsulation are presented in Table 2.

Spray drying remains the most industrially relevant method due to its scalability, cost efficiency, and compatibility with powdered food products; however, its high operating temperatures may compromise heat- and oxygen-sensitive minor VOCs if not carefully optimised. In contrast, freeze drying and electrospinning operate under milder conditions and generally offer superior retention of volatile compounds, although they require higher energy and have lower throughput.

Lipid-based systems such as nanoemulsions and liposomes provide effective solubilisation of REO and enable controlled release, making them particularly suitable for liquid foods, functional formulations, and enhancing bioavailability. Inclusion complexes with cyclodextrins offer efficient odour masking and improved water dispersibility but are limited by relatively low loading capacity. Polymer-based approaches, including ionic gelation, complex coacervation, and edible films, combine mild processing conditions with prolonged release profiles and are especially attractive for active food packaging applications.

From a functional perspective, encapsulation techniques that minimise thermal and oxidative stress such as freeze drying, ionic gelation or electrospinning are generally more effective at preserving the synergistic VOC network responsible for REO’s antioxidant and antimicrobial activities. Future research should therefore prioritise hybrid encapsulation systems, formulation optimisation, and process–structure–function relationships, aiming to balance industrial scalability, VOC preservation, and performance in real food matrices.

## 6. Potential Applications of Encapsulated REO in the Food Industry

Encapsulated REO has gained increasing relevance in the food industry as a natural preservative, functional ingredient, and component in active packaging. Examples of encapsulated REO used in real-world food matrices to extend shelf life are shown in Table 3.

Its antioxidant and antimicrobial properties, combined with the enhanced stability and controlled release provided by encapsulation, make it a valuable alternative to synthetic additives. Further potential applications of encapsulated REO in the food industry, demonstrating biological activity, are presented in Table 4.

### 6.1. Food Additive Preservation

Encapsulated REO can be used as a natural preservative to extend the shelf life of a variety of foods. Its strong antioxidant properties contribute significantly to the inhibition of lipid oxidation in high-fat matrices such as meat, poultry, dairy products and edible oils [3,87]. In addition, REO exhibits strong antimicrobial activity and effectively suppresses the growth of spoilage and pathogenic microorganisms on fresh produce, processed meat and bakery products. In addition to microbial control and oxidative stability, encapsulated REO also supports the preservation of important quality attributes such as colour, texture and nutritional value, maintaining the sensory and functional integrity of food products throughout storage.

The effects of REO encapsulation using whey protein isolate and inulin as encapsulation matrix on the shelf-life extension of Minas frescal cheese were evaluated [9]. The results showed that REO was effective in delaying microbial proliferation, particularly by controlling mesophilic bacterial growth, thereby extending the shelf life of the cheese without compromising its sensory properties. Edible coatings made from potato peel-derived polysaccharides, enriched with encapsulated REO and ascorbic acid, effectively enhanced the shelf life and quality of fresh-cut potatoes [79]. Coated samples, particularly those using the starch–pectin matrix and electrospun encapsulation, showed significantly reduced browning, microbial growth, and enzymatic activity over 14 days of storage. These findings highlight the potential of combining edible coatings with encapsulated bioactive agents as a sustainable and efficient strategy for preserving fresh produce and reducing food waste. Nanoemulsions formulated with REO exhibited strong antimicrobial activity in pork patties [77]. The synergistic effect between REO and oregano EO further enhanced preservation, allowing pork patties to be stored for up to 35 days under aerobic refrigeration conditions. Also, extremely low doses of REO in nanoemulsion mixed with cheese inhibited microbial growth and improved sensory properties [57].

### 6.2. Active Packaging Systems

Encapsulated REO is increasingly used in edible films, coatings and food packaging due to its multifunctional protective properties. Its antimicrobial activity provides surface protection by inhibiting the growth of spoilage and pathogenic microorganisms, thus extending shelf life and increasing food safety [5]. Encapsulation facilitates the controlled and prolonged release of REO’s bioactive compounds, maintaining efficacy over a longer period and reducing the need for synthetic preservatives. In addition, the encapsulated REO improves the barrier properties of packaging materials by reducing the permeability to oxygen and moisture, thus slowing down oxidative degradation and dehydration of food [88]. This multifunctional application meets the growing demand for sustainable, biodegradable and health-promoting packaging solutions in the food industry. REO was effectively incorporated into chitosan and chitosan-caseinate coatings on PLA films [89]. These REO-enriched films significantly reduced lipid oxidation, microbial spoilage and the formation of volatile spoilage markers in fresh chicken breast mince during refrigerated storage. Similar studies have been done by incorporating REO in chitosan/gelatine-based films [90]. The addition of REO increased the antioxidant activity of the films by about 60% compared to the control, while the high transparency was maintained and the mechanical strength was improved by about 30%. These results suggest that REO-enriched biopolymer films can serve as effective natural preservatives and provide a sustainable alternative to synthetic packaging materials by extending the shelf life of food products without compromising optical or structural integrity. This strategy is especially promising for sustainable and eco-friendly packaging that supports the shift away from synthetic preservatives.

### 6.3. Powdered Foods

Encapsulated REO, especially in spray-dried powder form, holds great potential for future use in instant soups, seasoning mixes, salad dressings, food supplements, beverage mixes and bakery premixes—product categories where thermal stability and extended shelf life are critical. Encapsulated REO was used in a salad dressing, where it showed strong antimicrobial activity against *S. aureus* and *E. coli* [91]. Although such applications are still under development, the encapsulated form offers promising benefits, including uniform distribution, controlled release during processing and better retention of antioxidant and antimicrobial activity compared to free EO.

## 7. Safety and Regulatory Framework of REO

In the European Union, the safety and regulatory framework for REO in the food industry is primarily based on the General Food Law and specific regulations on food additives, flavourings, and food supplements [92]. The General Food Law sets out the fundamental principles of food safety, including traceability, hygiene, and contaminant control, and assigns the European Food Safety Authority (EFSA) a central role in scientific risk assessment. REO intended for food use must meet strict quality criteria to ensure it is pure, natural, and free from adulteration with synthetic substances or other oils; this is verified through physicochemical tests (such as density, optical rotation, and refractive index) and chromatographic profiling. Although EOs as such are not authorised as food additives in the EU, many of their volatile constituents (e.g., eucalyptol, limonene, linalool) are approved as flavouring substances under Regulation (EC) No. 1334/2008 and are listed in the EU flavourings database. In the United States, the Food and Drug Administration (FDA) classifies REO as “Generally Recognized as Safe” (GRAS) when used at levels typical for food applications [93].

For encapsulated systems, safety considerations extend beyond the EO itself to the composition and structure of the wall material. Encapsulating agents should be selected from GRAS-listed substances, particularly when the final product is intended for direct human consumption. However, uncertainties remain regarding the long-term safety of encapsulated EOs, especially in nano-sized delivery systems. While nanotechnology offers promising strategies to enhance stability and bioavailability of bioactive compounds across different sectors, sub-micron materials may exhibit physicochemical properties distinct from their bulk counterparts, raising potential toxicological concerns [94].

The ingestion of higher doses of EO can cause serious oral toxicity. It is necessary to find a balance between the effective EO dose and the risk of toxicity [95]. Therefore, further clinical and toxicological studies are required to define safe daily intake levels of encapsulated REO for long-term consumption and to clarify their mechanisms of interaction within the human body. On the other hand, when encapsulated REO is incorporated into active packaging systems or edible films, its concentration must be carefully regulated to ensure sufficient biological activity (e.g., antimicrobial or antioxidant effects) while avoiding excessive migration or exposure levels that could compromise consumer safety. Also, in the EU, Directive 2003/89/EC requires that food product labels clearly declare the ingredients used in active packaging.

## 8. Research Gaps and Future Trends in the Application of Encapsulated REO

Future developments in food preservation and formulation increasingly indicate the replacement of synthetic antioxidants and antimicrobials with natural alternatives, driven by clean-label demands, sustainability concerns, and evolving regulatory frameworks. In this context, REO is among the most promising natural preservative systems due to its well-documented antioxidant and antimicrobial activities. Encapsulation technologies are expected to play a central role in this transition by addressing the inherent limitations of EOs, such as volatility, instability, and strong sensory impact, while preserving bioactivity at low effective concentrations.

A critical and currently underexplored research gap is the limited characterisation of REO composition after encapsulation. Most studies focus primarily on encapsulation efficiency and particle morphology, while far fewer systematically analyse how encapsulation processes affect the qualitative and quantitative VOC profile of REO. Changes in the relative abundance of major and minor constituents during encapsulation or storage may significantly alter synergistic interactions and biological activity. Future research should therefore place greater emphasis on post-encapsulation chemical profiling, using advanced analytical techniques such as GC–MS, GC–FID, or compound-specific isotope analysis, to link encapsulation conditions with the preservation of chemical integrity and functional performance. In addition, prior to large-scale production and commercialisation, the potential impact of REO on sensory attributes—particularly aroma and taste—must be systematically evaluated to ensure consumer acceptance. Well-designed sensory studies involving both trained panels and consumers are therefore essential.

A key research trend is moving beyond simple encapsulation efficiency towards a functionality-oriented design, in which encapsulation systems are tailored to preserve minor VOCs, maintain synergistic interactions, and control release kinetics under real food-processing and storage conditions. Greater emphasis on process–structure–function relationships, standardised evaluation protocols, and validation in complex food matrices will be essential to bridge the gap between laboratory-scale studies and industrial implementation.

Another emerging trend is the use of encapsulated REO as a dual-function ingredient, providing both aroma and biological activity. Unlike conventional flavourings that serve only a sensory role, encapsulated REO can deliver controlled aroma release while simultaneously exerting antioxidant and antimicrobial effects that enhance shelf life and functional quality. This dual functionality is particularly attractive for products such as sauces, dressings, bakery items, dairy foods, seasoning blends, and ready-to-eat formulations, where flavour perception, stability, and clean-label positioning are closely interconnected.

Overall, the future of encapsulated REO lies in its integration into multifunctional food systems, where aroma, preservation, and bioactivity are combined within a single ingredient. Advances in encapsulation technology, together with a deeper mechanistic understanding of VOC interactions and concentration-dependent effects, are expected to accelerate the adoption of REO and other EOs as a natural alternative to synthetic additives in next-generation food products.

## Figures and Tables

**Table 1:** Main bioactive components of REO reported in the literature.

Number of Samples	Origin of Plant	Plant Part Used	Extraction Method	Major Components	Analytical Method	References
6	Tunisia	leaves	Hydrodistillation	1.8-Cineole (47.2–27.5%) Camphor (12.9–27.9%)	GC-MS	[16]
6	Victoria (Australia), Alabama (USA), Western Cape (South Africa), Kenya, Nepal, and Yemen.	leaves	Hydrodistillation, Steam distillation	α-pinene (13.5–37.7%), 1,8-cineole (16.1–29.3%), Verbenone (0.8–16.9%)	GC-MS	[14]
3	Jordan	leaves	Supercritical fluid extraction	α-Pinene (44.4–55.1%), Camphene (8.0–11.8%), 1,8-Cineole (23.5–31.7%)	GC-MS	[17]
33	Israel	fresh leaves and stems, germplasm	Hydrodistillation	α-pinene (21.7–9.0%), 1,8-cineole (36.4–9.4%), verbenone (25.7–1.6%)	GC-MS	[18]
120	Italy, Spain	leaves	Organic extraction	α-Pinene, Camphor, 1,8-Cineole	GC-MS	[19]
1	Tunisia	leaves and twigs	Hydrodistillation	1,8-cineole (37%), camphor (12.5%), α-pinene (11.6%)	GC-MS	[20]
2	Turkey	leaves	Microwave-assisted Hydrodistillation	1,8-cineole, camphor, α-pinene	GC/MS, GC/FID	[21]
1	Malaysia	leaves	Hydrodistillation	α-pinene, and 1,8-cineole	GC–MS	[22]
1	Montenegro	leaves and twigs	Hydrodistillation	camphor (31.9%), borneol (12.2%), 1,8-cineole (11.3%)	GC/MS, GC/FID	[23]
4	Brazil	leaves	Vacuum fractional distillation	α-pinene (37.26%), 1,8-cineole (26.24%), verbenone (5.53%)	GC/MS, GC/FID	[24]
10	Balkan peninsula (Europe)	dried plant material	Hydrodistillation	1,8-cineole (10.9–39.4%), camphor (2.4–28.1%), borneol (2.1–21.4%), a-pinene (8.2–26.7%)	GC/FID, GC/MS.	[6]

**Table 2:** Comparative assessment and decision criteria for REO encapsulation techniques.

Encapsulation Technique	Thermal Stress	VOC Retention	EE ^1^	Release Profile	Scalability	Cost Level	Best-Suited Applications	Key Limitations
Spray Drying	High (short exposure)	Moderate–High (parameter-dependent)	High (optimised emulsion)	Diffusion-controlled, moderate	Very High (industrial)	Low	Powdered foods, seasoning mixes, dairy powders	Possible loss of minor thermolabile VOCs; surface oil formation
Freeze Drying	Minimal	High (preserves thermolabile compounds)	Moderate–High	Fast release (porous matrix)	Low	High	Active packaging, high-value nutraceuticals	High porosity; energy intensive
Micro- and nano-emulsions	Minimal	High (if protected from oxidation)	High	Diffusion-based, rapid bioavailability	Medium	Medium	Beverages, dairy systems, preservation-additive	Surfactant dependence; physical instability (Ostwald ripening)
Inclusion Complexation (Cyclodextrins)	Minimal	Selective retention (compound-specific affinity)	Moderate	Controlled; affinity-dependent	Medium	Medium	Odour masking, dry formulations	Limited loading capacity
Liposomes/Nanoliposomes	Low–Moderate	High (bilayer protection)	Moderate–High	Controlled, temperature-responsive possible	Medium–Low	High	Active packaging films, fresh produce preservation	Aqueous instability; cost
Ionic Gelation (Alginate-based)	Minimal	High	High	Sustained, matrix erosion-based	Medium	Low–Medium	Beverage inclusions, packaging beads	Particle size control
Complex Coacervation	Minimal	Very High	Very High	Strong controlled release	Medium	Medium	Targeted delivery systems	Batch variability
Electrospinning	Minimal	High	High	Slow diffusion, surface-area dependent	Low	High	Active edible films, antimicrobial coatings	Limited industrial scalability
Edible Films (Casting)	Minimal	Moderate–High	Moderate–High	Surface-release dominant	High	Low	Active packaging	Water sensitivity of films

^1^ EE—Encapsulation efficiency.

**Table 3:** Applications of REO in real-world food matrices.

Food Matrice	Dose of REO	Technological/Sensory Effect of REO	References
Minas Cheese	0.5% in product	Extended shelf life, controlled proliferation of mesophilic bacteria	[9]
Beef Hamburgers	1% in base mixture	Delayed lipid oxidation, sensory panel had great acceptance for hamburgers with REO	[76]
Pork Patties	2% in pork emulsion	Effectively inhibited oxidative and microbial spoilage of pork patties, enabling them to be stored successfully for 35 days at 4 °C in aerobic conditions, product was sensorially acceptable	[77]
Beef cutlet	0.0005–0.002% in nanogel	Reduce bacterial growth and extended shelf life	[78]
Cream and Karish cheese	1 × 10^−6^% in product	Inhibited microbial growth, addition of REO nano-emulsion significantly improved the sensory properties of cream, including aroma, colour, taste, texture, and overall acceptability	[57]
Potato	5% in starch-pectin coating	Reduced browning, enzyme activity, weight loss, hardness, and microbial load	[79]
Fresh Dough	1.5% in pure and encapsulated form	Product retained freshness encapsulated EO exhibited higher inhibition of fungi	[80]
Tomato juice	0.0015 and 0.0013% in product	Capsules were stable during tomato juice pasteurisation and maintained their antimicrobial activity	[81]
Strawberries	2% in coating	Extend the shelf life of the fruits	[82]
Queso Guoda cheese	5 or 10% in antimicrobial film	Prolonged shelf life of the product stored under refrigerated conditions	[70]

**Table 4:** Comparison of different encapsulation techniques and potential applications in food industry.

Encapsulation Technique	Wall Material	REO Load (%)	Application	Biological Activity	Characterisation of Capsules or Films	References
Spray drying	Maltodextrin, Modified starch	10–34.14	Functional food	/	MC, WS, D, DSC, SEM, EE	[49]
Edible films	Gelatin, chitosan, pectin	25	Food packaging, edible films	Antioxidant Antimicrobial	FTIR	[74]
Freeze drying	Whey protein isolate, Maltodextrin	4	Active packaging	Antibacterial	EE	[55]
Freeze drying	High amylose corn starch	5–10	Preservative in food	Antibacterial	TGA, XD, FTIR, DLS	[83]
Edible films	Gelatin	2	Edible film	Antioxidant Antimicrobial	/	[84]
Electrospinning	Zein, Zein nanofibres	1–10	Preservative in cheese	Antimicrobial	SEM, FTIR, TGA, EE	[70]
Electrospinning	Zein nanofibres, κ-carrageenan	0.5–50	Active food packaging	Antioxidant Antimicrobial	SEM, FTIR, DSC, MP	[71]
Nanoliposomes	Lecithin, Cholesterol, Polyvinylpyrrolidone, Tween 80	12.5	Protection of solid food	Antioxidant	EE, FTIR, SEM, AFM, XD, P	[64]
Liposomes	Liposome suspension, P90G	0.005–0.02	Preservation	Antioxidant Antimicrobial, Anti-inflammatory	DLS, ELS, TEM, EE	[85]
Liposomes	Liposome suspension	0.00005–0.0001	Enhancing shelf life for vegetables	Antioxidant	/	[86]
Ionic/crosslinking gelation	Calcium alginate, montmorillonite	1–3	Active food packaging	Antioxidant	FTIR, TGA, SEM, EE	[65]
Electrospinning, Spray drying, Freeze drying	Whey protein isolate, pullulan	5	Prolong shelf-life, increases storage stability	/	FTIR, EE, SEM, DSC	[56]
Nanoemulsion	Chitosan	20	Enhances physical meat quality, prevention of lipid oxidation	Antioxidant	SEM	[58]

## Data Availability

The original contributions presented in this study are included in the article. Further inquiries can be directed to the corresponding author.

## Abbreviations

The following abbreviations are used in this manuscript:

AFM	Atomic Force Microscopy
CD	Cyclodextrin
D	Density
DLS	Dynamic Light Scattering
DSC	Differential Scanning Calorimetry
EE	Encapsulation Efficiency
ELS	Electrophoretic Light Scattering
EO	Essential oil
FTIR	Fourier Transform Infrared Spectroscopy
MS	Moisture Content
MP	Mechanical Properties
NLC	Nanostructured Lipid Carriers
P	Porosity
REO	Rosemary Essential Oil
SEM	Scanning Electron Microscope
TEM	Transmission Electron Microscope
TGA	Thermogravimetric Analysis
VOC	Volatile Organic Compound
WS	Wettability And Solubility
XD	X-Ray Diffraction
